# Generation of Subwavelength Plasmonic Nanovortices via Helically Corrugated Metallic Nanowires

**DOI:** 10.1038/srep13089

**Published:** 2015-08-17

**Authors:** Changming Huang, Xianfeng Chen, Abiola O. Oladipo, Nicolae C. Panoiu, Fangwei Ye

**Affiliations:** 1 Department of Physics and Astronomy, Shanghai Jiao Tong University, Shanghai 200240, China; 2 IFSA Collaborative Innovation Center, Shanghai Jiao Tong University, Shanghai 200240, China; 3 Department of Electronic and Electrical Engineering, University College London, Torrington Place, London WC1E7JE, UK; 4 Bio-Nano Consulting, The Gridiron Building, One Pancras Square, N1C 4AG, London, United Kingdom

## Abstract

We demonstrate that plasmonic helical gratings consisting of metallic nanowires imprinted with helical grooves or ridges can be used efficiently to generate plasmonic vortices with radius much smaller than the operating wavelength. In our proposed approach, these helical surface gratings are designed so that plasmon modes with different azimuthal quantum numbers (topological charge) are phase-matched, thus allowing one to generate optical plasmonic vortices with arbitrary topological charge. The general principles for designing plasmonic helical gratings that facilitate efficient generation of such plasmonic vortices are derived and their applicability to the conversion of plasmonic vortices with zero angular momentum into plasmonic vortices with arbitrary angular momentum is illustrated in several particular cases. Our analysis, based both on the exact solutions for the electromagnetic field propagating in the helical plasmonic grating and a coupled-mode theory, suggests that even in the presence of metal losses the fundamental mode with topological charge *m* = 0 can be converted to plasmon vortex modes with topological charge *m* = 1 and *m* = 2 with a conversion efficiency as large as 60%. The plasmonic nanovortices introduced in this study open new avenues for exciting applications of orbital angular momentum in the nanoworld.

Optical vortices are light beams characterized by a phase change of an integer multiple of 2*π* along a closed path around the center of the beam, where the phase of the beam is undetermined (phase singularity) and the field amplitude vanishes^1^,^2^,^3^. The interest in such optical structures has dramatically increased since a connection between the Laguerre-Gaussian laser modes and the orbital angular momentum (OAM) of light has been established^4^. In particular, it has been demonstrated that these beams and other optical vortices carry an OAM of *mħ* per photon^4^, where *m* is the so-called topological charge of the optical vortex. This discovery has spurred intensive research interest as in addition to its impact at a more fundamental science level, it has been realized that OAM-carrying optical vortices could find a series of appealing applications to optical tweezers^5^,^6^, optical spectroscopy^7^, digital imaging^8^, and quantum information processing^9^,^10^. Importantly, significant advances in this field have been facilitated by the fact that vortex beams can be readily generated by using a multitude of experimental setups, including mode converters by astigmatic lenses^11^,^12^, computer-synthesized holograms^10^,^13^, spiral-phase plates^14^, angular gratings^15^, and twisted elliptical^16^ and photonic crystal fibers^17^,^18^. One drawback of these methods, which severely hinders the extension of the applications of optical vortices to the nanoscale, is that the diffraction limited propagation of the vortical beams generated by these methods leads to spatially delocalized optical beams with size significantly larger than the operating wavelength.

A recently introduced, promising approach towards optical beam engineering at subwavelength scale, relies on plasmonic metasurfaces consisting of planar nanopatterned metallic structures. In particular, thin metallic films in which nanoapertures with various shapes were milled in^19^,^20^,^21^,^22^ or planar arrays of metallic nanoantennae^23^ were used to experimentally generate plasmonic vortices. Despite the fact that the phase of plasmonic vortices generated by these techniques can vary at subwavelength scale, their size, however, was still much larger than the operating wavelength. As an effective solution to this problem, in this report we demonstrate that one can generate subwavelength optical vortices by first confining the optical field to subwavelength scale using a metallic nanowire, the highly localized optical mode being then converted into an optical vortex by means of a helical grating imprinted on the surface of the nanowire. Importantly, the generation of subwavelength optical beams with zero angular momentum by using metallic nanowires has been investigated both theoretically^24^,^25^ and experimentally^26^, whereas the optical modes of helical gratings made of perfect conductors have been studied in a recent theoretical work^27^. Our theoretical and computational study presented in this paper suggests that these ideas can be extended to the generation of subwavelength optical vortices, namely one can employ plasmonic helical gratings to convert the fundamental plasmonic mode of a uniform metallic nanowire to an optical beam carrying OAM, the conversion efficiency being as large as 60% even in the presence of optical losses in the metal.

## Results

### Plasmonic helical grating

**Figure 1:**
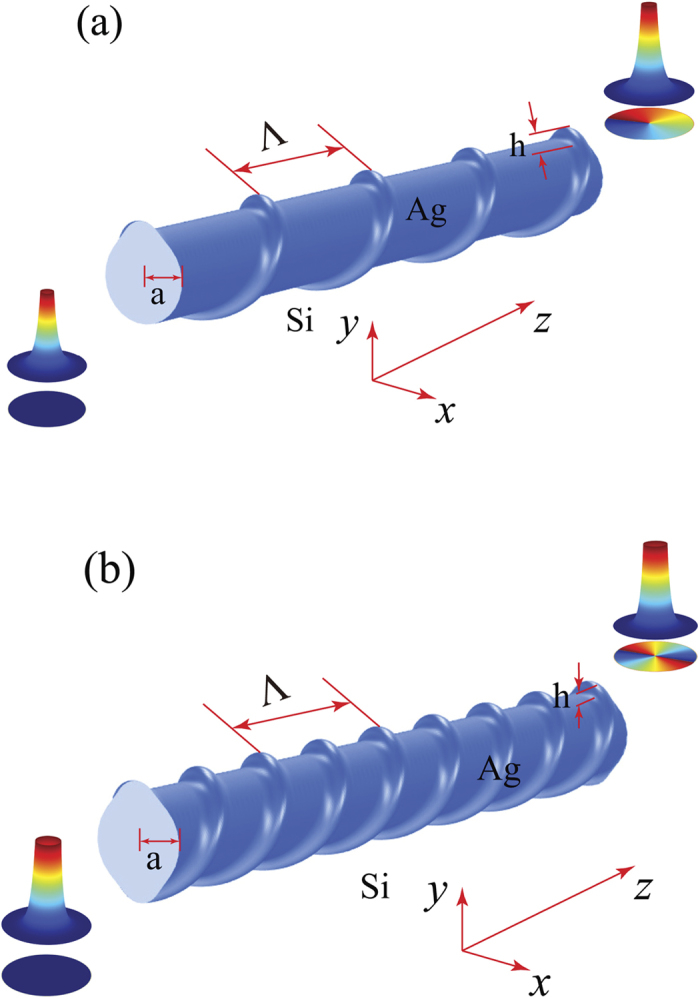
Schematic of a metallic (Ag) nanowire with a single and double helical surface grating. The helical surface gratings are designed to convert the fundamental mode with topological charge *m* = 0 to a plasmon vortex mode with topological charge *m* = 1 (**a**) and *m* = 2 (**b**).

The plasmonic helical grating designed to convert optical modes of a uniform metallic nanowire is schematically depicted in Fig. 1. It consists of a metallic cylinder with constant radius, *a*, the surface of the cylinder being engraved with a helical periodic grating with period, Λ, and height, 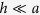. Although it is a challenging feat, the nanofabrication of such plamsonic helices has been recently reported in several works^28^,^29^. In particular, rotating the ion beam of a focused-ion beam system at high speed while periodically changing the radius of beam rotation^30^ or using metal-assisted chemical etching^31^,^32^, helical nanostructures similar to those investigated in this work could be fabricated. We assume that the metallic nanowire is embedded in a dielectric medium, the choice of materials considered in this paper being silver and silicon, respectively. When a helical grating is imprinted on the nanowire, the fundamental plasmon mode, which can be excited by an incident TM-polarized Gaussian beam, can be converted into vortex modes provided that the wavevector mismatch between the fundamental mode and the desired vortical mode is compensated by the properly designed grating.

### Mode analysis of the uniform metallic nanowire

The physical characteristics of the mode conversion process depend on the properties of the optical modes of the uniform (constant transverse section) metallic nanowire as well as the geometrical and electromagnetic properties of the plasmonic helix. Regarding the optical modes of the nanowire, the main quantities that determine the mode conversion efficiency are the field distribution and the modal propagation constant. In the case of uniform metallic nanowires the optical modes can be readily obtained analytically in cylindrical coordinates, *r*, *ϕ*, and *z* (see Supporting Information). For convenience, we denote the optical modes as 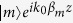, where *β*_*m*_ is the effective refractive index of the mode and *k*_0_ = *ω*/*c* is the wavenumber in vacuum at the carrier frequency, *ω*. The quantum number, 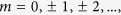 also called topological charge, defines the order of the mode and also describes the dependence of the optical field on the azimuthal angle, via the exponential factor *e*^*imϕ*^.

**Figure 2:**
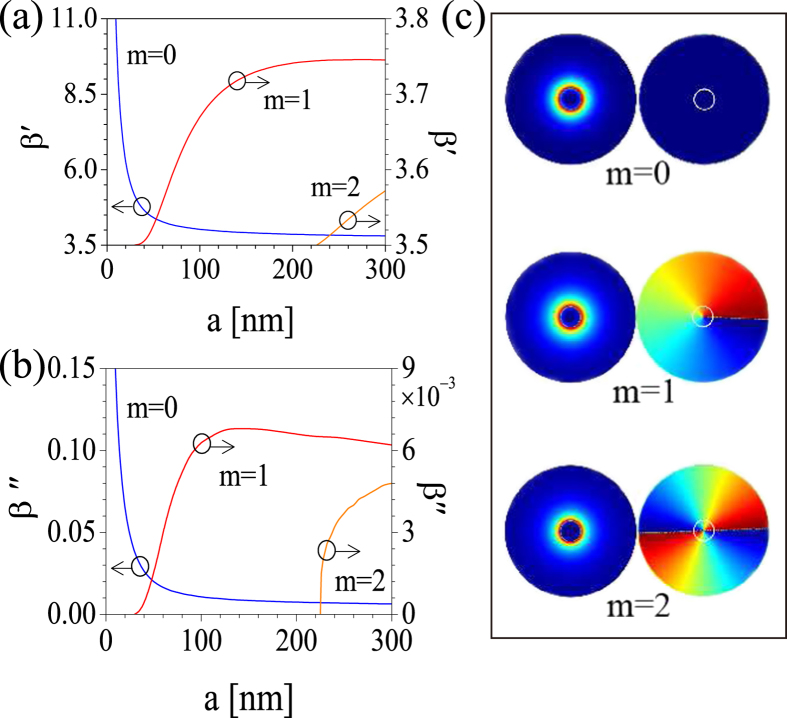
Dispersion and field profiles of optical modes of metallic nanowires. (**a**,**b**) Real and imaginary part of the effective mode index for the first three modes, respectively. (**c**) Spatial profile of the amplitude and phase of the modes with *m* = 0, *m* = 1, and *m* = 2. In all panels, *λ* = 1500 nm.

The results of our mode analysis are presented in Fig. 2, where we plot the variation of effective mode refractive index vs. the radius of the metallic nanowire, *a*, as well as the modal amplitude and phase distributions, all determined for the first three modes, *m* = 0, 1, 2. In this work we use the convention that the sign of the topological charge is positive (negative) if the phase increases clockwise (counterclockwise) around the phase singularity. Figure 2 shows that the fundamental mode does not have a cut-off frequency, namely it exists for any radius of the nanowire, whereas for a given frequency vortical modes can only be supported by a nanowire if its radius is larger than a certain critical value. Moreover, this cut-off radius increases as the topological charge of the vortex mode increases. Note that the cut-off radii of the nanowire corresponding to the vortices with charge *m* = 1 and *m* = 2 are still well within the subwavelength regime, which suggests that metallic nanowires could potentially be used to generate subwavelength optical vortices. Another important feature of the optical modes of the nanowire is revealed by Fig. 2(b), namely their propagation loss dispersion. Specifically, the propagation loss of the fundamental and vortical plasmon modes, which is proportional to 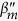, has contrasting dependence on the nanowire radius: while in the case of the fundamental mode the propagation loss decreases sharply with the radius, in the case of vortical modes there is a steep increase with the radius near the mode cut-off, which is followed by a region in which the propagation loss decreases slowly with the radius. Here, *z*′ (*z*″) represents the real (imaginary) part of the complex number *z*. In particular, at *λ* = 1500 nm the figure of merit of plasmon modes, defined as 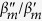, is ~10^–2^ and ~10^–3^ for the fundamental mode and vortices, respectively.

### Coupled-mode theory

The phase-matching condition for efficient mode conversion can be readily derived by using a vectorial coupled-mode theory (CMT). In the standard framework of CMT, the total electromagnetic field in the perturbed nanowire (helical grating) is expressed as linear super-position of the modes of the uniform nanowire, 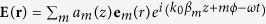 and 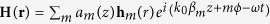, where **e**_*m*_(*r*) and **h**_*m*_(*r*) are the electric and magnetic fields of the mode with topological charge, *m*, of the unperturbed nanowire, respectively. The main result provided by the CMT is the coupled-mode equations (CMEs), which govern the dependence of the mode amplitudes, *a*_*m*_(*z*), on the propagation distance (see Supporting Information for the derivation of these equations):


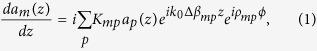


where 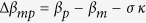 and 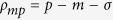 quantify the phase mismatch between modes 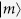 and 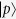, in the longitudinal and transverse directions, respectively, the reduced wave vector, 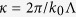, and *σ* is the order of the helix, namely the order of the symmetry point group *C*_*n*_ of the helix (e.g., *σ* = 1 and *σ* = 2 for a single- and a double-helix, respectively). The coefficients *K*_*mp*_ describe the coupling between the two interacting modes, and is given by the following overlap integral:


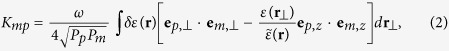


In this equation *P*_*i*_ is the normalization power of mode 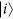, the dielectric perturbation *δε*(**r**) is the difference between the dielectric constant of the uniform nanowire and helical grating, 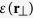 and 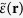 are the dielectric constant of the uniform nanowire and helical grating, respectively, and the symbol “
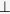
” indicates the transverse component of a vector.

It can be clearly seen from Eq. (1) that in order to achieve an efficient energy transfer between the modes 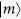 and 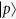, their longitudinal and transverse phase-mismatch must be simultaneously compensated by a proper choice of the helical perturbation, *δε*(**r**). To be more specific, we assume that the dielectric constant of the plasmonic system can be expressed as follows:


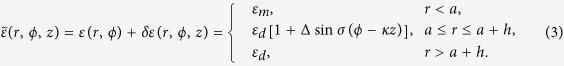


The parameters Δ and *κ* represent the grating perturbation strength and the grating wave vector, respectively. Specifically, Eq. (3) describes a helix imprinted onto the background region (chosen to be silicon in our case, *ε*_*d*_ = 12.25) within a cylindrical shell with thickness, *h*. Similarly, the helix can be imprinted into the metallic region (silver in our case, *ε*_*m*_ = −125 at *λ* = 1500) as well. We have investigated both cases, the main conclusions being qualitatively similar.

As explained above, efficient mode conversion occurs provided that the grating wave vector, *κ*, and the helix order, *σ*, are chosen so as both the longitudinal and transverse phase difference between the two interacting modes are compensated. For example, in order to convert the fundamental mode (*m* = 0) into a vortex mode with charge *m*, the pitch of the helix must satisfy the relation 
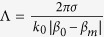
 and the order of the helix should be *σ* = *m*. Importantly, the handedness of the helical grating determines the sign of the generated vortex. This can be easily understood by expanding the perturbation as 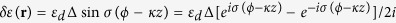. As the nanowire excitation mode is 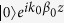, the only two vortices that could be excited via the helical grating described by Eq. (3) are 
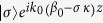
 and 
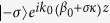
. However, the latter vortex, which has a negative charge, has a larger effective mode index as compared to that of the fundamental mode and therefore it is not phase-matched to the excitation mode. Therefore, it is expected that in this case only the vortex with positive charge, *m* = *σ*, is generated. On the other hand, if the handedness of the helix is reversed, a negatively charged vortex would be generated.

**Figure 3:**
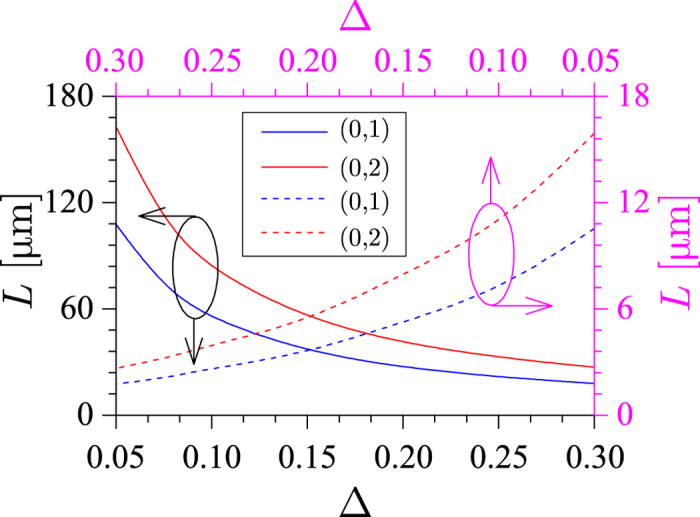
Coupled-mode theory analysis of mode interaction. Coupling length between the fundamental mode and the vortex modes with charge *m* = 1 (blue lines) and *m* = 2 (red lines). The grating is imprinted into the Si background (solid lines) and onto the Ag nanowire (dashed lines). System parameters are *λ* = 1500 nm and *h* = 20 nm. Λ = 5.03 μm and *a* = 110 nm (Λ = 5.09 μm and *a* = 250 nm) for the 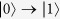 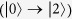 mode conversion process.

A key quantity that characterizes the mode conversion process is the conversion length, *L*, which is defined as the distance over which the fundamental mode is completely converted into a vortex mode 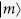. It can be easily shown that Eq. (1) implies that the conversion length is given by 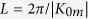, which suggests that a larger optical coupling between the interacting modes should lead to a shorter conversion length. This conclusion is fully supported by the results presented in Fig. 3, where we plot the dependence of the coupling length on the grating perturbation strength, Δ, for the mode conversion processes 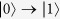 and 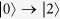. This figure shows that the coupling length decreases when the grating strength, Δ, increases, which suggests that it could be possible to reach an operation regime in which the coupling length is smaller than the characteristic loss length of the interacting modes by simply increasing the grating strength. Moreover, it can be seen that the coupling length is larger when the grating is imprinted into the silicon background as compared to the case when it is engraved onto the metallic nanowire. This is an expected result as in the latter case there is a larger perturbation of the dielectric constant of the system, due to the fact that the dielectric constant of silver is significantly larger than that of silicon. In addition, for both types of helical gratings, the coupling length increases with the charge of the generated vortex, chiefly due to the fact that the overlap between the fundamental mode and the vortex modes decreases with the topological charge.

### Rigorous numerical simulations

Encouraged by these results derived from the CMT, we sought to validate them by using rigorous electromagnetic numerical simulations based on the fully 3D exact solutions of the Maxwell equations. To this end, we determined first from the CMT the pitch, Λ, of the helical grating by imposing the condition that the two interacting optical modes are phase-matched. Then, we launched the fundamental mode (*m* = 0) at the input facet of a plasmonic helical grating designed to phase-match this mode and a specific vortical mode (*m* ≠ 0), the total 3D field distribution being determined by numerically solving the Maxwell equations^33^. As an alternative to this rigorous approach, we used Eq. (1) to calculate the amplitudes of the interacting modes and, subsequently, the 3D field distribution. As a result, this computational analysis would provide valuable insights into the regime in which the predictions of the CMT are valid.

**Figure 4:**
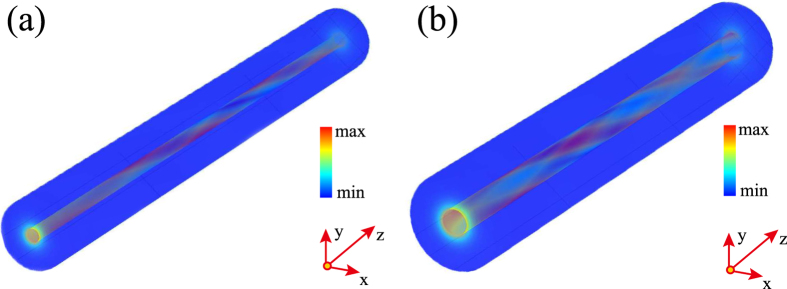
Field distributions obtained by solving the 3D Maxwell equations. The numerical simulations were performed for (**a**) a single- and (**b**) double-helix structure. The system parameters in the left (right) panel are Λ = 5.03 μm and *a* = 110 nm (Λ = 5.09 μm and *a* = 250 nm). In both cases *λ* = 1500 nm and *h* = 20 nm.

We have followed the computational approach just described and studied the vortex generation processes 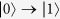 and 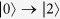, the corresponding field dynamics being presented in Fig. 4(a,b), respectively. These field profiles clearly illustrate the formation of optical vortices. In addition, they show an important feature of the mode conversion process, namely, unlike the case of helical optical fibers, the length over which the fundamental mode is converted into a vortex mode is larger but comparable to the pitch of the helix, Λ.

### Mode conversions

**Figure 5:**
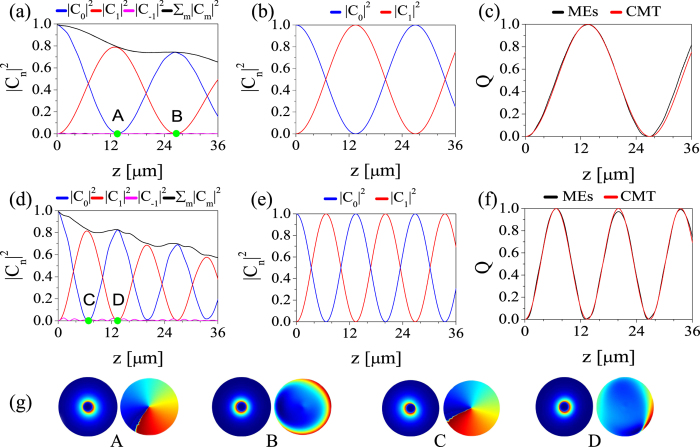
Generation of nanovortices with topological charge *m* = 1. Variation of mode weight, 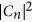, and effective topological charge, *Q*, vs. propagation distance, *z*, calculated for grating strength Δ = 0.1 (top panels) and Δ = 0.2 (middle panels). Results in panels (**a**,**d**) are found by solving the 3D Maxwell equations whereas those in panels (**b**,**e**) are calculated using the CMT. (**g**), The amplitude (left panels) and phase (right panels) structure of the plasmonic field, calculated at four distances, as shown in panels (**a**,**d**). Other parameters are: *λ* = 1500 nm, *h* = 20 nm, Λ = 5.03 μm, and *a* = 110 nm.

Let us now analyze more closely the 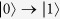 conversion process. The physical quantities most suitable for characterizing the efficiency of this process are the effective topological charge of the field, 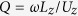, where the total orbital angular momentum of the mode, *L*_*z*_, and its intensity, *U*_*z*_, are given by


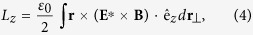



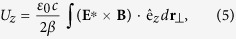


and the modal weight, *C*_*m*_, defined as


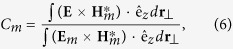


which quantifies the relative amount of power flowing in the mode, *m*. The dependence of these physical quantities on the propagation distance, *z*, is presented in Fig. 5. We considered two helical gratings with values of the grating strength, namely Δ = 0.1 (top panels) and Δ = 0.2 (middle panels). As the figure shows, upon the propagation of the fundamental mode in the grating, its weight, 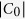, gradually decreases with *z*, whereas the weight of the 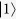 vortex, 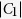, increases until the maximum conversion is reached at a quarter of coupling length, *z* = *L*/4. The maximum mode conversion corresponds to the point *A* (*C*) in Fig. 5(a) [Fig. 5(d)]. The corresponding intensity and phase distribution at these points are shown in Fig. 5(g). Because the mode interaction increases with the grating strength, the coupling length should decrease with Δ [compare the location of points *A* and *C* in Fig. 5(a) and Fig. 5(d), respectively]. This is the expected dynamics of the plasmonic field indeed, as the grating was designed to phase match the interaction of the 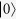 and 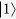 modes. Beyond the maximum mode conversion point the power flow between the two modes is reversed. Note, however, one interesting idea revealed by Fig. 5(d): the sum of mode powers weakly increases whenever the transformation of the vortex mode into the fundamental mode occurs, indicating that some energy of the radiation modes is fed back into the nanostructure during the back-conversion.

During the mode conversion, as expected, the effective topological charge of the total field, *Q*, displays the same evolution as 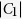, approaching almost unit value at the coupling length [Fig. 5(c,f)]. Note that, due to its phase-mismatched with the fundamental or other modes, the mode 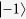, namely the vortex with charge *m* = −1, is not excited. It should be noted that, however, due to the excitation of radiative modes and possibly other vortical modes with larger topological charge during the mode conversion, not all of the energy of the fundamental mode is transferred to the 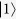 vortex, thus both 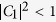 and *Q* < 1 at *z* = *L*. Despite this, a remarkably large conversion efficiency can be achieved, more than 80% of the energy of the fundamental mode being transferred to the 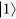 vortex. We also studied the plasmonic field evolution and the corresponding physical quantities that characterize its dynamics by using the CMT, the results being presented in Fig. 5(b,e). One can see that the predictions of the CMT regarding the coupling length are in very good agreement with the results of direct simulations. As expected, the conversion efficiency calculated using the CMT agrees less with the simulation results, primarily because we included in the CMT calculations only the two interacting modes.

**Figure 6:**
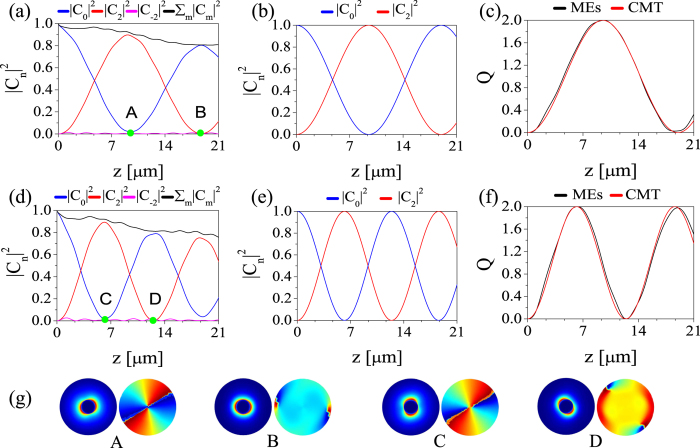
Generation of nanovortices with topological charge *m* = 2. Variation of mode weight, 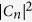, and effective topological charge, *Q*, vs. propagation distance, *z*, calculated for grating strength Δ = 0.2 (top panels) and Δ = 0.3 (middle panels). Results in panels (**a**,**d**) are found by solving the 3D Maxwell equations whereas those in panels (**b**,**e**) are calculated using the CMT. (**g**), The amplitude (left panels) and phase (right panels) structure of the plasmonic field, calculated at four distances, as shown in panels (**a**,**d**). Other parameters are: *λ* = 1500 nm, *h* = 20 nm, Λ = 5.09 μm, and *a* = 250 nm.

Our analysis shows that a single-helical metallic nanowire with length *L* can be viewed as a source of unit-charge nanovortices, the radius of the generated vortices being roughly equal to the radius of the nanowire. In the case of Fig. 5, vortices have a radius of about 110 nm, which is more than an order of magnitude smaller than the operating wavelength, *λ* = 1500 nm. Similarly, metallic nanowires with double-helix surface corrugation, that is *σ* = 2 in Eq. (3), could be used to generate optical vortices with topological charge equal to 2. This is clearly demonstrated by the plots presented in Fig. 6, which summarize the results of our analysis of the field dynamics in a double-helix plasmonic grating designed to phase-match the fundamental mode and the 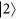 vortex. However, it should be mentioned that, the higher the order of the desired vortex is, the larger its cut-off value for a specific radius will be. This suggests that the size of the generated vortices increases with the order of the vortex. Despite this, the generated doubly-charged vortex shown in Fig. 6 has a radius of 250 nm, which is still significantly smaller than the wavelength.

**Figure 7:**
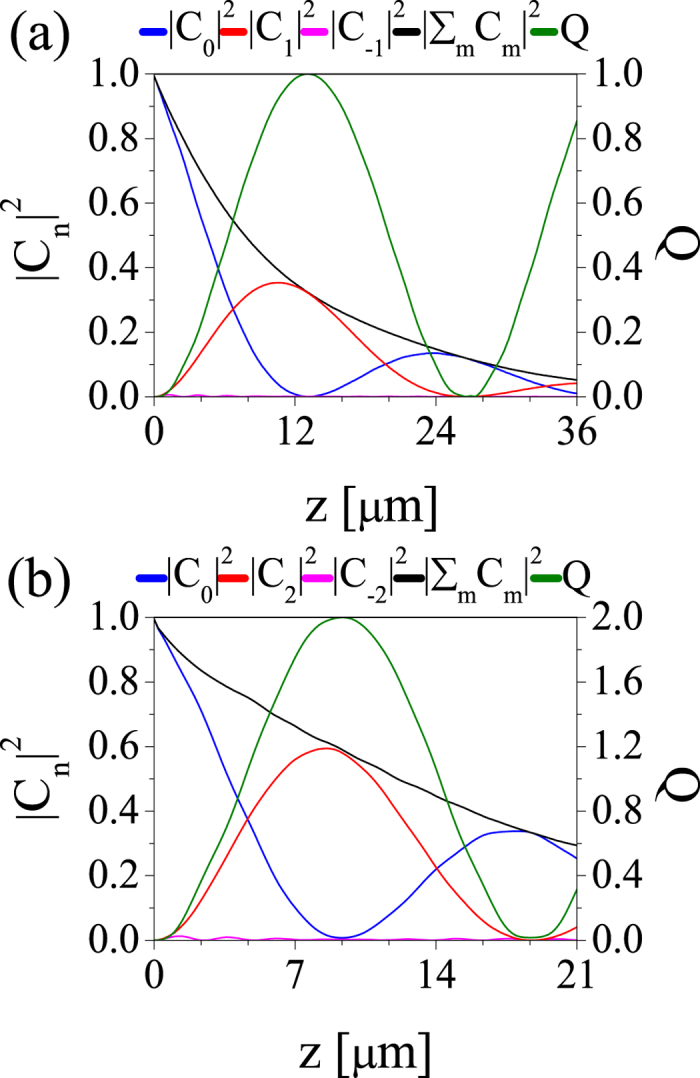
Generation of optical vortices with charge *m* = 1 (a) and *m* = 2 (b) in lossy metallic helical gratings. The upper (lower) panel corresponds to the case presented in the top panels of Fig. 5 (Fig. 6).

Finally, we considered the influence of the metal losses on the generation of nanovortices. Metallic losses can be particularly detrimental to the vortex generation process as the electromagnetic energy could be dissipated over an effective loss length that is smaller than the coupling length, so that no vortices would be generated. Fortunately, one can decrease the coupling length by simply increasing the grating strength, Δ, or by increasing its height, *h* [see Fig. 3; also compare the coupling lengths in Fig. 5(a,d) or those in Fig. 6(a,d)], so that one can easily achieve significant mode conversion before the fundamental mode decays. The conversion of the fundamental mode into the 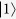 and 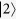 nanovortices, when metallic losses are fully incorporated in simulations, are shown in Fig. 7(a,b), respectively. This figure suggests that a surprisingly large mode conversion efficiency can be achieved, namely ~35.3% and ~60.2% for the vortices with topological charge *m* = 1 and *m* = 2, respectively. The fact that lower efficiency is achieved in the case of the vortex with *m* = 1 can be explained by noticing that the optical field of this vortex is more confined around the metallic nanowire and therefore the corresponding optical losses are larger.

## Conclusions

In conclusion, in this study we have introduced a new type of sources of nanovortices, namely, metallic cylinders with deep-subwavelength radius and helically corrugated surfaces. With a proper selection of the period of the helix, these helical gratings can be used to generate nanoscale vortices with various topological charge. A coupled-mode theory of mode conversion was developed, its predictions being in excellent agreement with the conclusions of direct simulations based on the full set of Maxwell equations. The plasmonic nanovortices introduced in this study might extend a series of appealing applications of OAM-carrying light beams to the nanoworld, such as nanoscaled optical spanners^5^ and digital imagining^8^, as well as the integrated quantum information processing^9^.

## Additional Information

**How to cite this article**: Huang, C. *et al.* Generation of Subwavelength Plasmonic Nanovortices via Helically Corrugated Metallic Nanowires. *Sci. Rep.* **5**, 13089; doi: 10.1038/srep13089 (2015).

## Supplementary Material

Supplementary Information
