# Supplementary material for: Generation of Subwavelength Plasmonic Nanovortices via Helically Corrugated Metallic Nanowires
Source: Sci Rep. 2015 Aug 17;5:13089. doi: 10.1038/srep13089 (PMC4538392; doi:10.1038/srep13089)
Supplement: Supplementary Information [file srep13089-s1.pdf]

Supporting Information: Generation of Subwavelength Plasmonic Nanovortices via Helically Corrugated Metallic Nanowires

Changming Huang,[1] Xianfeng Chen,[1] Abiola O. Oladipo,[2] Nicolae C. Panoiu,[2] and Fangwei Ye[1]

[1]*State Key Laboratory on Advanced Optical Communication Systems and Networks, Department of Physics and Astronomy, Shanghai Jiao Tong University, Shanghai 200240, China*

[2]*Department of Electronic and Electrical Engineering, University College London, Torrington Place, London WC1E7JE, UK*

S1. Optical modes of metallic cylinders

We consider an infinite metallic cylinder of radius, a, embedded in a dielectric medium, the axis of the cylinder being oriented along the z-axis [see Fig. 1(a)]. The permittivity of the metallic cylinder and dielectric background is assumed to be ε_m and ε_d, respectively. Solving Maxwell equations in the frequency domain in cylindrical coordinates (r, ϕ, z) leads to analytical expressions for the electric and magnetic fields. Apart from a common exponential factor, $e^{i(k_0\beta_m z+m\phi-\omega t)}$, these fields are given by:

$$e_z(r) = C_1 I_m(\gamma_1), \tag{1a}$$

$$h_z(r) = D_1 I_m(\gamma_1), \tag{1b}$$

$$e_r(r) = -\frac{ik_0 r}{\gamma_1^2}\left[m(C_1\beta_m + iD_1 Z_0)I_m(\gamma_1) + C_1\beta_m\gamma_1 I_{m+1}(\gamma_1)\right], \tag{1c}$$

$$h_r(r) = -\frac{ik_0 r}{\gamma_1^2}\left[m\left(D_1\beta_m - iC_1\frac{\varepsilon_m}{Z_0}\right)I_m(\gamma_1) + D_1\beta_m\gamma_1 I_{m+1}(\gamma_1)\right], \tag{1d}$$

$$e_\phi(r) = -\frac{ik_0 r}{\gamma_1^2}\left[m(iC_1\beta_m - D_1 Z_0)I_m(\gamma_1) - D_1 Z_0\gamma_1 I_{m+1}(\gamma_1)\right], \tag{1e}$$

$$h_\phi(r) = -\frac{ik_0 r}{\gamma_1^2}\left[m\left(iD_1\beta_m + C_1\frac{\varepsilon_m}{Z_0}\right)I_m(\gamma_1) + C_1\frac{\varepsilon_m}{Z_0}\gamma_1 I_{m+1}(\gamma_1)\right], \tag{1f}$$

for $r \le a$, where $\gamma_1 = k_0 r\sqrt{\beta_m^2 - \varepsilon_m}$, and

$$e_z(r) = C_2 K_m(\gamma_2), \tag{2a}$$

$$h_z(r) = D_2 K_m(\gamma_2), \tag{2b}$$

$$e_r(r) = -\frac{ik_0 r}{\gamma_2^2}\left[m(C_2\beta_m + iD_2 Z_0)K_m(\gamma_2) - C_2\beta_m\gamma_2 K_{m+1}(\gamma_2)\right], \tag{2c}$$

$$h_r(r) = -\frac{ik_0 r}{\gamma_2^2}\left[m\left(D_2\beta_m - iC_2\frac{\varepsilon_d}{Z_0}\right)K_m(\gamma_2) - D_2\beta_m\gamma_2 K_{m+1}(\gamma_2)\right], \tag{2d}$$

$$e_\phi(r) = -\frac{ik_0 r}{\gamma_2^2}\left[m(iC_2\beta_m - D_2 Z_0)K_m(\gamma_2) + D_2 Z_0\gamma_2 K_{m+1}(\gamma_2)\right], \tag{2e}$$

$$h_\phi(r) = -\frac{ik_0 r}{\gamma_2^2}\left[m\left(iD_2\beta_m + C_2\frac{\varepsilon_d}{Z_0}\right)K_m(\gamma_2) - C_2\frac{\varepsilon_d}{Z_0}\gamma_2 K_{m+1}(\gamma_2)\right], \tag{2f}$$

for $r \ge a$, where $\gamma_2 = k_0 r\sqrt{\beta_m^2 - \varepsilon_d}$.

In these equations $Z_0 = \sqrt{\mu_0/\varepsilon_0}$ is the impedance of the vacuum, $I_m(x)$ and $K_m(x)$ denote the modified Bessel functions of the first and the second kind, respectively, and the constants C_1, C_2, D_1, and D_2 are related to each other via the boundary conditions at $r = a$. These boundary conditions also yield a transcendental equation for the propagation constant of the mode, β_m:

$$\left[1 + \frac{\bar\gamma_1(\beta_m^2 - \varepsilon_d)}{m(\varepsilon_m - \varepsilon_d)}\frac{I_{m+1}(\bar\gamma_1)}{I_m(\bar\gamma_1)} + \frac{\bar\gamma_2(\beta_m^2 - \varepsilon_m)}{m(\varepsilon_m - \varepsilon_d)}\frac{K_{m+1}(\bar\gamma_2)}{K_m(\bar\gamma_2)}\right]\left[1 + \frac{\bar\gamma_1\varepsilon_m(\beta_m^2 - \varepsilon_d)}{m(\varepsilon_m - \varepsilon_d)\beta_m^2}\frac{I_{m+1}(\bar\gamma_1)}{I_m(\bar\gamma_1)} + \frac{\bar\gamma_2\varepsilon_d(\beta_m^2 - \varepsilon_m)}{m(\varepsilon_m - \varepsilon_d)\beta_m^2}\frac{K_{m+1}(\bar\gamma_2)}{K_m(\bar\gamma_2)}\right] = 1.$$

where $\bar\gamma_1 = k_0 a\sqrt{\beta_m^2 - \varepsilon_m}$ and $\bar\gamma_2 = k_0 a\sqrt{\beta_m^2 - \varepsilon_d}$.

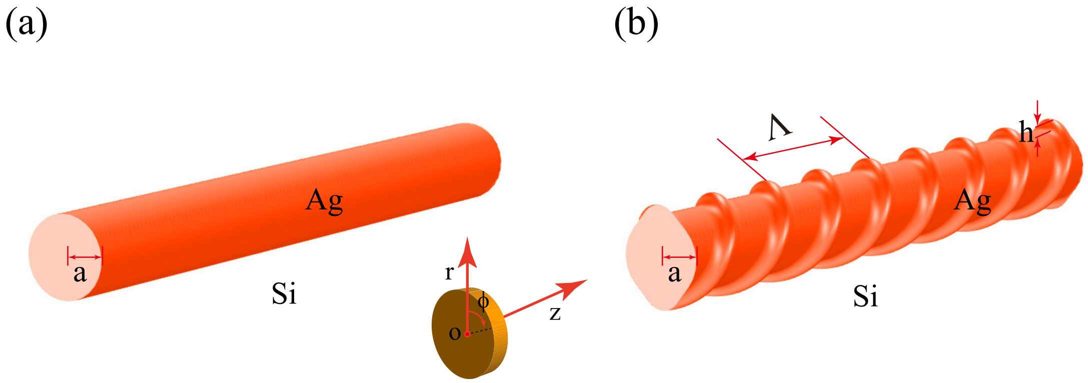

FIG. 1. (a) A uniform metallic nanowire with a radius a, and (b) a surface-helically-corrugated metallic nanowire with pitch, Λ, and height of the corrugation, h.

S2. Derivation of the coupled-mode equations

In the framework of the coupled-mode theory, the total electric and magnetic fields, $\mathbf{E}(\mathbf{r})$ and $\mathbf{H}(\mathbf{r})$, respectively, of the perturbed (helically corrugated) plasmonic structure are expanded as linear superposition of the normal modes of an unperturbed nanowire [1]:

$$\mathbf{E}_\perp(\mathbf{r}_\perp, z) = \sum_n \frac{a_n(z)}{\sqrt{P_n}} \mathbf{e}_\perp^{(n)}(\mathbf{r}_\perp), \tag{3a}$$

$$E_z(\mathbf{r}_\perp, z) = \sum_n \frac{a_n(z)}{\sqrt{P_n}} \frac{\varepsilon(\mathbf{r}_\perp)}{\tilde{\varepsilon}(\mathbf{r})} e_z^{(n)}(\mathbf{r}_\perp), \tag{3b}$$

$$\mathbf{H}(\mathbf{r}_\perp, z) = \sum_n \frac{a_n(z)}{\sqrt{P_n}} \mathbf{h}_\perp^{(n)}(\mathbf{r}_\perp), \tag{3c}$$

where $a_n(z)$ is the mode amplitude of the n-th mode, measured in units of $\mathrm{W}^{1/2}$, $\varepsilon(\mathbf{r}_\perp)$ and $\tilde{\varepsilon}(\mathbf{r})$ are dielectric constant of the unperturbed and perturbed nanowire, respectively, and $\mathbf{e}^{(n)}(\mathbf{r}_\perp)$ and $\mathbf{h}^{(n)}(\mathbf{r}_\perp)$ are the electric and magnetic fields of the n-th mode. The longitudinal translational invariance of the unperturbed plasmonic structure implies that the electromagnetic field of the modes depends only on the transverse coordinate, $\mathbf{r}_\perp$. Each optical mode is normalized so that $\int_S [\mathbf{e}_n \times \mathbf{h}_m^* + \mathbf{e}_m^* \times \mathbf{h}_n] \cdot \hat{\mathbf{z}} dS = 4P_n\delta_{nm}$, where P_n is the optical power carried by the n-th mode.

To find the mode amplitudes, one starts from the unconjugated form of the Lorentz reciprocity theorem [2],

$$\frac{\partial}{\partial z}\int_S [\mathbf{E}_1(\mathbf{r},\omega) \times \mathbf{H}_2(\mathbf{r},\omega) - \mathbf{E}_2(\mathbf{r},\omega) \times \mathbf{H}_1(\mathbf{r},\omega)] \cdot \hat{\mathbf{z}} dS = i\omega \int_S [\epsilon_2(\mathbf{r}) - \epsilon_1(\mathbf{r})]\mathbf{E}_1(\mathbf{r},\omega) \cdot \mathbf{E}_2(\mathbf{r},\omega) dS, \tag{4}$$

where $(\mathbf{E}_1, \mathbf{H}_1)$ and $(\mathbf{E}_2, \mathbf{H}_2)$ are solutions of the Maxwell equations corresponding to the dielectric constants $\epsilon_1(\mathbf{r})$ and $\epsilon_2(\mathbf{r})$, respectively. As fields $(\mathbf{E}_1, \mathbf{H}_1)$, one chooses the n-th, backward $(-z)$ propagating mode, whereas for the fields $(\mathbf{E}_2, \mathbf{H}_2)$ one chooses those given by eqs 3, namely,

$$\epsilon_1(\mathbf{r}) \equiv \epsilon(\mathbf{r}_\perp) \tag{5a}$$

$$\mathbf{E}_1(\mathbf{r}) = \frac{1}{\sqrt{P_m}}[\mathbf{e}_{m,\perp}(r) - \mathbf{e}_z(r)]e^{-i(\beta_m z + m\phi)} \tag{5b}$$

$$\mathbf{H}_1(\mathbf{r}) = \frac{1}{\sqrt{P_m}}[-\mathbf{h}_{m,\perp}(r) + \mathbf{h}_z(r)]e^{-i(\beta_m z + m\phi)} \tag{5c}$$

$$\epsilon_2(\mathbf{r}) \equiv \epsilon(\mathbf{r}_\perp) + \delta\epsilon(\mathbf{r}) \tag{5d}$$

$$\mathbf{E}_{\perp,2}(\mathbf{r}) = \sum_p \frac{a_p(z)e^{i(\beta_p z + p\phi)}}{\sqrt{P_p}} \mathbf{e}_{p,\perp}(r) \tag{5e}$$

$$\mathbf{E}_{z,2}(\mathbf{r}) = \sum_p \frac{a_p(z)e^{i(\beta_p z + p\phi)}}{\sqrt{P_p}} \frac{\epsilon(\mathbf{r}_\perp)}{\epsilon_2(\mathbf{r})} \mathbf{e}_z(r) \tag{5f}$$

$$\mathbf{H}_2(\mathbf{r}) = \sum_p \frac{a_p(z)e^{i(\beta_p z + p\phi)}}{\sqrt{P_p}} \mathbf{h}_p(r) \tag{5g}$$

where we have expressed the permittivity of the helical nanowire [Fig. 1(b)], $\epsilon_2(\mathbf{r})$, as the sum of the permittivity of the unperturbed nanowire and the helical perturbation: $\epsilon_2(\mathbf{r}) = \epsilon_1(\mathbf{r}_\perp) + \delta\epsilon(\mathbf{r})$. The helical perturbation can be expanded in Fourier series as $\delta\epsilon(\mathbf{r}) = \sum_\sigma \delta\epsilon_\sigma(r) e^{-i\sigma(\phi+\kappa z)}$, where $\kappa = \frac{2\pi}{k_0\Lambda}$ and Λ is the pitch of the helix. This series expansion shows that the helix can impart onto the propagating plasmonic field both an additional longitudinal momentum as well as an azimuthal angular momentum.

Inserting the fields given by eqs 5 into the l.h.s. of Lorentz reciprocity theorem, eq 4, one obtains:

$$\frac{\partial}{\partial z}\int_S (\mathbf{E_1}\times\mathbf{H_2} - \mathbf{E_2}\times\mathbf{H_1})\cdot\hat{z}dS = \frac{\partial}{\partial z}\int_S \sum_p \frac{a_p(z)e^{i(\beta_p z+p\phi)}}{\sqrt{P_p P_m}}(\mathbf{e}_m\times\mathbf{h}_p + \mathbf{e}_p\times\mathbf{h}_m)\cdot\hat{e}_z dS e^{-i(\beta_m z+m\phi)}$$

$$= 4\frac{d}{dz}\sum_p A_{mp}a_p(z)e^{i[(\beta_p-\beta_m)z+(p-m)\phi]} = 4\frac{da_m(z)}{dz}, \tag{6}$$

where $A_{mp} = \frac{1}{4\sqrt{P_p P_m}}\int_S (\mathbf{e}_m\times\mathbf{h}_p + \mathbf{e}_p\times\mathbf{h}_m)\cdot\hat{e}_z dS$. Note that the normalization condition, $A_{mp} = \delta_{mp}$, has been used to derive eq 6. Moreover, substituting eqs 5 into the r.h.s. of eq 4, one finds,

$$i\omega\int_S (\epsilon_2(\mathbf{r}) - \epsilon_1(\mathbf{r}))\mathbf{E}_1\cdot\mathbf{E}_2 dS = 4i\sum_p K_{mp}a_p(z)e^{i[(\beta_p-\beta_m)z+(p-m)\phi]}, \tag{7}$$

where

$$K_{mp} = \frac{\omega}{4\sqrt{P_p P_m}}\iint \delta\epsilon(\mathbf{r})\left[\mathbf{e}_{p,\perp}\mathbf{e}_{m,\perp} - \frac{\epsilon(\mathbf{r}_\perp)}{\tilde{\epsilon}(\mathbf{r})}\mathbf{e}_{p,z}\mathbf{e}_{m,z}\right]dS. \tag{8}$$

In eq 8, $\tilde{\epsilon}(\mathbf{r}) \equiv \epsilon_1(\mathbf{r}_\perp) + \delta\epsilon(\mathbf{r})$. Note that the value of K_{mp} can be easily found by simply evaluating numerically the double integral in the equation above.

Finally, assuming that the order of the helix is σ, one can derive the system of equations describing the dynamics of the mode amplitudes,

$$\frac{da_m(z)}{dz} = i\sum_p K_{mp}a_p(z)e^{ik_0\Delta\beta_{mp}z}e^{i\rho_{mp}\phi}, \tag{9}$$

where $\Delta\beta_{mp} = \beta_p - \beta_m - \sigma\kappa$ and $\rho_{mp} = p - m - \sigma$.

[1] F. Ye, D. Mihalache, B. Hu, and N. C. Panoiu, *Phys. Rev. Lett* **104**, 106802 (2010).
[2] A. W. Synder and J. D. Love, *Optical Waveguide Theory*, (Chapman and Hall, London, 1983).
